# Impact of stigma on the placement of mental health facilities: insights from early career psychiatrists worldwide

**DOI:** 10.3389/fpsyt.2023.1307277

**Published:** 2023-12-18

**Authors:** Leila Kamalzadeh, Renato de Filippis, Samer El Hayek, Mohsen Heidari Mokarar, Chonnakarn Jatchavala, Eugene Boon Yau Koh, Amine Larnaout, Isa Multazam Noor, Margaret Isioma Ojeahere, Laura Orsolini, Mariana Pinto da Costa, Ramdas Ransing, Mohammad Amin Sattari, Mohammadreza Shalbafan

**Affiliations:** ^1^Geriatric Mental Health Research Center, Department of Psychiatry, School of Medicine, Iran University of Medical Sciences, Tehran, Iran; ^2^Psychiatry Unit, Department of Health Sciences, University Magna Graecia of Catanzaro, Catanzaro, Italy; ^3^Medical Department, Erada Center for Treatment and Rehabilitation in Dubai, Dubai, United Arab Emirates; ^4^Department of Psychiatry, Imam Hossein Hospital, School of Medicine, Alborz University of Medical Sciences, Karaj, Iran; ^5^Department of Psychiatry, Faculty of Medicine, Prince of Songkla University, Songkhla, Thailand; ^6^Department of Psychiatry, Faculty of Medicine and Health Sciences, Universiti Putra Malaysia, Serdang, Selangor, Malaysia; ^7^Faculty of Medicine, University of Tunis El Manar, Tunis, Tunisia; ^8^Department of Psychiatry D, Razi Hospital, Tunis, Tunisia; ^9^Department of Psychiatry, Faculty of Medicine, YARSI University, Jakarta, Indonesia; ^10^Child and Adolescent Mental Health Unit, Dr Soeharto Heerdjan Teaching Mental Hospital, Jakarta, Indonesia; ^11^Department of Psychiatry, Jos University Teaching Hospital, Jos, Plateau State, Nigeria; ^12^Unit of Clinical Psychiatry, Department of Neurosciences/Department of Experimental and Clinical Neurosciences (DIMSC), Polytechnic University of Marche, Ancona, Italy; ^13^Institute of Psychiatry, Psychology and Neuroscience, King's College London, London, United Kingdom; ^14^Institute of Biomedical Sciences Abel Salazar, University of Porto, Porto, Portugal; ^15^Department of Psychiatry, Addiction Medicine, and Clinical Neurosciences, All India Institute of Medical Sciences, Guwahati, Assam, India; ^16^Mental Health Research Center, Psychosocial Health Research Institute (PHRI), Department of Psychiatry, School of Medicine, Iran University of Medical Sciences, Tehran, Iran

**Keywords:** mental health, mental disorders, psychiatry, art, social stigma, health facilities, psychiatric hospital

## Introduction

In the realm of mental health care, the placement and organization of facilities have long been intrinsically linked to prevailing societal and cultural attitudes and the persistent stigma surrounding mental illness (1). Psychiatric hospitals, known as “asylums”, were often located in remote areas due to safety concerns, driven in part by misconceptions and stigma (2). The mid-twentieth century marked the emergence of the deinstitutionalization movement, aimed to reintegrate patients with mental illnesses into the community by placing mental health facilities within or close to urban areas and providing community mental health care (3). Despite these positive movements, traces of stigma continue to influence the geographical positioning and structure of mental care facilities (4). We synthesized the viewpoints of some Early Career Psychiatrists (ECPs) Section members from the World Psychiatric Association (WPA) (5), who were within the age bracket of 30–47 years. Our inquiry aimed to examine the influence of stigma on the location and configuration of mental health establishments, as well as its effects on the professional identities and levels of job satisfaction among psychiatrists. This exploration spanned across 10 distinct national contexts, including: India, Indonesia, Iran, Italy, Lebanon, Malaysia, Nigeria, Thailand, Tunisia, and the United Kingdom, as summarized in Tables 1A, B. We also provided recommendations for improving the quality and accessibility of mental health care.

**Table 1A:** Presence and location of psychiatric facilities and stigma.

**Country**	**Presence of psychiatric hospitals**	**Location of psychiatric hospitals**	**Specific location of psychiatric wards in general hospitals**	**Stigma influence**	**Stigmatizing terms**
India	Yes	Large cities	Yes	Significant	“Mental,” “Psycho,” “Pagal”
Indonesia	Yes	Countryside	Yes	Significant	“Crazy”
Iran	Yes	Countryside	Yes	Significant	“Timarestan,” “Divaneh,” “Ravani”
Italy	No, closed in 1978	-	Yes	Previously high, now lower although present	“Manicomio” (asylum), “Ospedale dei pazzi o dei matti” (hospital of the fools), “Madhouse”
Lebanon	Yes	Formerly isolated, now central	No	Significant	“Majnoun,” “Akhwat”
Malaysia	Yes, integrated	Within city hospitals	Yes	Significant	“Tanjung Rambutan”
Nigeria	Yes	Large Cities	Yes	Significant but improving	“Yaba left”
Thailand	Yes	Large Cities	Yes	Variable	None
Tunisia	Yes	City centers	No	Previously high, now low	Famous stigmatizing terms
UK	Yes	Variable	Yes	Variable	“Asylum”

**Table 1B:** Programs, integration, and views on psychiatric care.

**Country**	**Benefits of remote locations**	**Destigmatization programs**	**Medical integration**	**Employment views**	**Deinstitutionalization**
India	No benefits	Training of primary care physicians and health care professionals	Can help reduce stigma	Mixed based on hospital	Supported
Indonesia	Dignity and privacy of patients	Expansion of Liaison-Consultant Psychiatry	Can help reduce stigma	Positive employment views	Supported
Iran	No benefits	Mental health education for health care workers and the public	Experience shows benefits	Mixed attitude	Supported but challenges remain
Italy	Not applicable	Considered (e.g., Establishment of youth-friendly hubs)	Experience shows it reduces stigma	Lingering stigma remains	Strongly supported
Lebanon	No benefits	Conducted by major hospitals and educational institutions, as well as non-governmental organizations	Helps reduce stigma	No employment effect	Supported
Malaysia	No benefits	No sustained anti-stigma program	No effect	No stigma effect	Supported
Nigeria	Reduced cost, easy access	Awareness programs	Helps reduce stigma	Mixed attitude	Supported but with caution regarding practicability, models, etc.
Thailand	Some benefits	Community-based psychiatric care (Village health volunteer training)	Helps reduce stigma	Mixed attitude	Caution adapting western models
Tunisia	Remoteness from the noise and the pollution	National programs, hospital closure advocated	Did not reduce stigma	No employment effect	Strongly supported
UK	None described	“Choose psychiatry” campaign	Potentially positive	Possible stigma effect	Not a priority currently

## Segregation in placement of psychiatric hospitals and wards

In most of the countries where we collected the views of psychiatrists, standalone psychiatric hospitals still comprise a significant portion of psychiatric beds, with diverse geographical distribution patterns influenced by factors such as population density, urbanization, and healthcare infrastructure. However, the specter of stigma noticeably affects the geographic placement of psychiatric facilities in various nations. For instance, Indonesia and Iran have predominantly located their psychiatric hospitals in the countryside. This choice stems from misconceptions about mental illness, with the assumption that remote locations with stringent security measures will prevent patients from wandering or posing threats to the community (6, 7). Conversely, in places like India, Tunisia, Lebanon, and Nigeria, psychiatric hospitals are predominantly situated in large urban centers. Malaysia initially constructed psychiatric hospitals in suburban areas but later shifted them to city centers with urban expansion. The UK and Thailand exhibit variable rural and urban placement patterns, while Italy underwent significant transformations due to anti-stigma efforts initiated by “Basaglia's Law” in 1978, resulting in the closure of psychiatric asylums (usually located in peripheral areas), the organization of psychiatric assistance through the creation of territorial psychiatry departments and the integration of psychiatry wards (with a limited number of bed seats) for the management of acute phases of psychiatric diseases, within general hospitals in medium and large cities (8).

On a more positive note, some psychiatrists highlighted potential benefits of locating psychiatric hospitals in areas distant from urban centers. These benefits include reduced exposure to urban noise and pollution and reduced transport costs for individuals residing in rural areas. Additionally, the case of the Aro Village Project in Nigeria was mentioned. This innovative initiative, led by Prof. Thomas Adeoye Lambo, established a community-based mental health care system that prioritized affordability, accessibility, and cultural appropriateness. The project involved villagers in the accommodation and treatment of psychiatric patients, integrating family members and traditional healers into the care process. This initiative transformed social perceptions of madness and evolved from a rural mental hospital into a community situated in an urban center (9).

Regarding the placement of psychiatric units or wards within general hospitals, over half of the respondents reported that psychiatric wards in their respective countries were situated in specific locations, such as separate buildings, lower floors, or isolated wings. These wards often feature specialized facilities, security measures, and unique ward architecture, including closed walls and security guards. While these security measures primarily aim to ensure patient safety and prevent suicides, this segregation inadvertently perpetuates stigma by communicating separation between physical and mental healthcare. Patients' restricted access to outdoor spaces due to security concerns fosters feelings of isolation and marginalization. Moreover, disparities in resource allocation are observed, with specialists from other fields often exhibiting a negative view or even fear of psychiatric patients. In some cases, these specialists refuse to accept patients with psychiatric disorders into their departments.

## Use of stigmatizing language

Many countries still use stigmatizing terms for psychiatric hospitals, wards, or locations. Terms like “crazy” and “psycho” label patients, while references to “asylums” further perpetuate institutional stigma. For example, “Tanjung Rambutan” is the location of Malaysia's first psychiatric hospital, but it is also used as a derogatory and discriminatory term for someone who is not accepted and should be locked up in an asylum. Similarly, some countries, like Thailand, consistently use “neuro” instead of “psycho” (or neuro-psycho) in official terms, hospitals, and institutes (10). Such stigmatizing terminology may reflect and reinforce societal prejudice (11).

## Integration of medical services

Opinions on the establishment of medical inpatient wards or outpatient clinics of various specialties, such as neurology, internal medicine, or emergency care, within psychiatric hospitals as means to reduce stigma diverged. Some believed that such integration reduced stigma by fostering frequent interactions between psychiatrists and non-psychiatry practitioners, challenging stereotypes and discrimination. However, others argued that stigma persisted across various fields, often stemming from professionals' own understanding of mental illness.

## Impact of stigma on employment choices

Perspectives varied on how stigma associated with psychiatric hospitals affects psychiatrists' employment choices. Some observed willingness among younger generations to foster positive change through such positions. However, stigma appears to deter others from these career paths. Improved infrastructure can increase desirability of psychiatric hospital employment. Overall, individual attitudes likely involve multiple factors like past training experiences and job availability.

## Implementation of destigmatization programs

Psychiatrists also highlighted the implementation of destigmatization initiatives in their regions. These efforts encompassed the expansion of Liaison-Consultant Psychiatry, the establishment of off-site centers for adolescents with mental distress, the training of primary care physicians and specialists from various fields, the introduction of CBT-based anti-stigma programs for caregivers of psychiatric patients, and initiatives to raise awareness about mental health issues among children, and youths. Additionally, community-based psychiatric care programs involving Village Health Volunteers (VHVs) were mentioned. However, despite these endeavors, the absence of comprehensive, sustainable, and long-term destigmatization programs, coupled with financial constraints and inadequate support from authorities, remains a significant challenge in this regard.

## Deinstitutionalization considerations

Respondents' opinions on the deinstitutionalization movement also vary. Many express supports for deinstitutionalization, citing reasons such as reducing stigma, increasing access to care, respecting patient preferences, enhancing family and social support, and saving costs. Some mention that modern mental health hospitals offering high-quality services may render deinstitutionalization less urgent. Others emphasize the importance of considering social welfare and contextual culture when implementing deinstitutionalization.

## Discussion

In summary, despite notable advancements in the deinstitutionalization movement and the incorporation of mental health care into mainstream healthcare systems, the enduring impact of stigma continues to shape the placement and structure of psychiatric facilities. It is crucial to acknowledge and actively address these persistent stigmatizing influences to ensure comprehensive and inclusive healthcare for individuals with psychiatric disorders, thereby enhancing their wellbeing and alleviating the societal burden of mental illness. Future research and policy endeavors should prioritize the dismantling of these barriers and the cultivation of a more compassionate and integrated approach to mental health care. A balanced strategy is recommended: (1) Foster the development of accessible community-based care by allocating adequate resources and implementing anti-stigma initiatives; (2) In cases where psychiatric hospitals are still deemed necessary, introduce internal destigmatization programs while concurrently devising transitional plans to gradually shift toward community-based models when sustainability allows.

## Author contributions

LK: Conceptualization, Data curation, Investigation, Project administration, Writing—original draft. RdF: Conceptualization, Data curation, Writing—review & editing. SE: Conceptualization, Data curation, Writing—review & editing. MH: Data curation, Writing—review & editing. CJ: Data curation, Writing—review & editing. EK: Data curation, Writing—review & editing. AL: Data curation, Writing—review & editing. IN: Data curation, Writing—review & editing. MO: Data curation, Writing—review & editing. LO: Data curation, Writing—review & editing. MP: Conceptualization, Data curation, Supervision, Writing—review & editing. RR: Data curation, Writing—review & editing. MAS: Data curation, Writing—review & editing. MS: Conceptualization, Data curation, Investigation, Project administration, Supervision, Writing—review & editing.
